# The Influence of Energy Balance, Lipolysis and Ketogenesis on Metabolic Adaptation in Cows Milked Twice and Three Times Daily

**DOI:** 10.3390/metabo12111090

**Published:** 2022-11-10

**Authors:** Srđan Krnjaić, Marko Cincović, Radojica Djoković, Branislava Belić, Jožica Ježek, Jože Starič

**Affiliations:** 1 Department of Veterinary Medicine, Faculty of Agriculture, Novi Sad, University of Novi Sad, Trg Dositeja Obradovića 8, 21000 Novi Sad, Serbia; 2 Department of Animal Science, Faculty of Agronomy Čačak, University of Kragujevac, Cara Dušana 34, 32000 Čačak, Serbia; 3 Veterinary Faculty, University of Ljubljana, Gerbičeva 60, 1000 Ljubljana, Slovenia

**Keywords:** cow, metabolic status, blood, milk, lactation

## Abstract

Increasing milk production requires increasing milking frequency (MF) from two times daily (2X) to three (3X) or more. High milk production leads to negative energy balance (NEB) and homeorhesis, characterized by lipolysis, ketogenesis, and endocrinological changes. The relationship among energy balance (EB), lipolysis, and ketogenesis with endocrine and metabolic parameters in blood of cows milked 2X and 3X daily was studied. Holstein Friesian cows milked 2X (*n* = 45) and 3X (*n* = 45) were analyzed, with approximately 50% of cows in each group in positive EB (PEB) and 50% in NEB. After determining EB, blood samples were collected from all cows and blood serum was analyzed for non-esterified fatty acids (NEFA), beta-hydroxybutyrate (BHB), glucose (GLU), cholesterol (CHOL), triglycerides (TGC), total bilirubin (TBIL), aspartate aminotransferase (AST), gamma-glutamyl transferase (GGT), calcium (Ca), inorganic phosphate (P), total protein (TPROT), albumin (ALB), urea, insulin (INS), T3, T4, and cortisol (CORT), and the RQUICKIBHB index of insulin resistance was calculated. Cows milked 3X in NEB represent a special cluster that partially overlaps with cows milked 2X in NEB and has no contact points with cows in PEB. Cows milked 3X had higher levels of NEFA, BHB, AST, GGT, TBIL, and CORT and lower levels of GLU, Ca, INS, and T4. Cows milked 3X in NEB had higher levels of NEFA, BHB, AST, GGT, TBIL, and CORT and lower levels of GLU, CHOL, TGC, TPROT, P, INS, RQUICKIBHB, and T3 compared with cows milked 2X in NEB and cows in PEB. In cows milked 3X, lipolysis and ketogenesis were much more prominent, and EB levels were lower, implying a pronounced shift in homeorhesis. Metabolic and endocrinology parameters were determined mainly by the values of EB, NEFA, and BHB in cows milked 3X in NEB compared with other categories of cows. The results confirm the peculiarity of metabolic adaptation in cows with increased MF, characterized not only by differences in the concentration of metabolites but also in their interactions.

## 1. Introduction

More than five decades ago, more frequent milking was found to have long-term effects on milk production [1]. With the development of automatic milking and increasing knowledge about the physiology of lactation, milking frequency (MF) began to increase. On most farms, cows are milked twice daily (2X). Due to the increase in milk production, farmers often decide to increase MF, so cows are milked three times daily (3X) or more. Increasing MF to 3X increases milk production by 6–26% throughout lactation [2,3,4]. A large positive shift in milk production due to increased MF appears to be constant, with daily milk production rising between 3.5 and 4.9 kg/day [5]. The effect of increased MF is most pronounced in early lactation (7–8 kg/day), and thereafter, the increase is smaller until the end of lactation (2.5–5.1 kg/day) [6,7].

Higher milk production should stimulate dry matter intake (DMI) due to increased energy and nutrient requirements. A previous study showed a moderate linear relationship (r^2^ = 0.47) between DMI and milk yield (MY), and models based on intake of nutrients improved prediction of MY in Holstein cows compared with DMI alone, so intake of NEL was the dominant variable in MY prediction models [8]. However, the results of studies show that cows milked 3X and 4X did not have increased DMI relative to cows that were milked less frequently [9,10,11] and that it was significantly higher only when the MF was increased to six milkings per day [12]. Genetic selection for high milk production is not associated with the ability of cows to have higher DMI, so high milk production predisposes cows to negative energy balance (NEB) [13]. Furthermore, adding energy to the feed of cows producing large amounts of milk tends to further increase milk production rather than decrease NEB [13]. Increased milk and energy-corrected milk (ECM) production can stimulate the development of NEB in cows, especially because there is a weak genetic correlation between ECM and DMI in cows, meaning that increases in ECM production are not accompanied by a corresponding increase in DMI [14,15]. Patton et al. [16] found that daily energy balance (EB) was less negative in cows on a standard diet milked once a day than in cows milked 3X and that they had lower MY and higher milk fat and milk protein concentrations during weeks 1 to 3 of lactation.

Cows milked 3X exhibited lower body condition, more pronounced lipolysis (higher non-esterified fatty acids, NEFA) and ketogenesis (higher beta-hydroxybutyrate, BHB), with an increase in growth hormone levels and a decrease in insulin concentration and slightly higher cortisol levels [6,11]. Reducing MF to once daily can decrease lipolysis and ketogenesis and improve the metabolic status of cows [17,18,19]. A recent study suggests that such a reduction may be a useful tool for treating ketosis in cows [20].

Metabolic adaptation to increasing MF corresponds to NEB of cows and homeorhesis in early lactation, which is characterized by increased lipolysis and ketogenesis with dominance of catabolic processes and the development of several unfavorable metabolic adaptations [21,22,23]. NEFA and BHB show significant correlations with parameters of metabolic profile, indicating the functional status of carbohydrate, fat, protein, mineral, and liver metabolism, as well as with hormones and indicators of cellular stress response in cows [24,25,26].

Previous studies have not investigated whether there is a difference in metabolic adaptation in cows in positive energy balance (PEB) and NEB at different milking frequencies. The aim of this study was to investigate the relationship between EB, lipolysis, and ketogenesis with endocrine and metabolic parameters in the blood of cows milked 2X and 3X daily in different phases of lactation.

## 2. Materials and Methods

### 2.1. Farm Management

The experiment was conducted on a commercial dairy farm with 700 Holstein Friesian cows housed in two identical free-stall barns located next to each other with a head-to-head system and central feeding corridor. The farm was in the transition from 2X to 3X milking during the study, so half of the cows were milked 2X and half 3X per day with an average MYs of 8100 and 9000 kg per standard lactation, respectively. Cows were housed in groups according to phase of lactation (LP). In each barn, cows were assigned as those in first and second half of lactation and as optimal or high productivity cows. The 2X and 3X milked cows were subjected to the same experimental design, with similar animal characteristics (DMI, MY, days in milk (DIM), parity, and environmental factors) within the group and the same total mixed ration (TMR) and identical pens.

The 2X and 3X groups were fed TMR twice daily (after morning and evening milking) or three times daily (after morning, midday, and evening milking), respectively (Table 1). The composition of the TMR was in accordance with NRC (2001) [27] recommendations, based on NRC feedstuff composition tables and specifications provided by feed additive and concentrate manufacturers. The data calculated were also compared with data obtained from analysis of the TMR using standard procedures and the *Van Soest* method using ANKOM fiber analyzer (ANKOM Technology, New York, NY, USA) to determine whether the TMR feed differed from what was expected from the tables. Water was available ad libitum.

On the farm, DMI was determined using a model. For each group of cows on the farm, the ration was composed according to their average production, body weight, and milk fat percentage. When preparing a ration, the DMI was first predicted, and then the ration was balanced. Therefore, our experiment was based on the predicted DMI using the formula: DMI (kg/d) = 0.372 × FCM (kg/d) + 0.0968 × BW (kg^0.75^) × [1-e (−0.192 × (WOL + 3.67))], where FCM = 4% fat-corrected milk (kg/d), metabolic BW = body weight (kg^0.75^), WOL = week of lactation, and d = day [27]. To be able to monitor feed consumption continually and relatively accurately, a suitable feed bunk scoring system was introduced on the basis of the method developed at Penn State University. In this system, points are assigned from 0 (when no feed is left in the feed bunk) to 5 (when no feed is taken at all). A score of 2 is considered optimal when less than 5% of the amount given at the last feeding remained in the feed bunk. The appropriate time for scoring is in the last hour before the next feeding [28]. There was at least 0.8 m of feed bunk space per cow in the experiment. No antagonistic or other behavior affecting feed intake was observed in the cows during the experiment.

### 2.2. Energy Balance

The EB was calculated from energy-corrected milk yield (ECM), NEL-intake (NEL–net energy of lactation), and BW using the following equation: EB = [NEL-intake − (kg ECM × 3.14 + 0.293 × kg BW^0.75^)] [29]. NEL-intake, BW, and kg ECM were determined during one week, and expressed as an average value (7 days average), before the sampling of blood on Day 7. NEL-intake depends on predicted DMI. The body weight was measured with a scale. Milk production was recorded by farm software. The chemical composition of milk was determined with MilkoScan analyser (FOSS, Hileroed, Denmark) using Fourier transform infrared spectrophotometry. Milk samples (40 mL) were collected according to the recommendations of the analyzer manufacturer, during regular milking. The content of fat, protein, lactose, and dry matter was determined. Before analysis, the samples were heated in a water bath to a temperature of 40 ± 2 °C. The analyzer homogenizes the milk sample before removing approximately 5 mL of it for analysis. ECM was calculated according to the formula: ECM = (0.327 × milk lbs.) + (12.95 × fat lbs.) + (7.65 × protein lbs.) [27].

### 2.3. Models and Cows Included in Experiment

This research explored the metabolic adaptations that occurred using a 2 × 2 × 3 factorial assignment of cows: milked 2X vs. 3X, negative vs. positive energy balance, and lactation stage (early vs. mid vs. late). Randomly selected cows in second and third lactation, in optimal body condition for their LP (mean BCS 3, range 2.75–3.5), and without clinical health problems (they were also not lame); milked 2X daily (milking interval 10–12 h) (N = 45) and 3X daily (milking interval 6–8 h) (N = 45) were enrolled in the study. The cows were in early (30–60 DIM), mid (120–190 DIM), and late lactation (220–290 DIM). Approximately 50% of the cows in each group were in PEB and 50% in NEB. Finally, the cows were categorized so that cows in different LP were approximately equally represented in the PEB and NEB groups (Table 2). The minimum number of cows enrolled in the study was determined based on the number of experimental groups, according to previously described methods [30]. Moreover, that number was increased to ensure adequate number of sampled animals that would allow us to obtain a statistically significant difference in metabolite values between the investigated groups. To calculate the number of animals needed, we used data on the variability of metabolic parameters determined in our previous research and an online sample size calculator: https://homepage.univie.ac.at/robin.ristl/samplesize.php (accessed on 22 April 2022), with targeted power = 0.8 and significance threshold alpha = 0.05.

### 2.4. Blood Metabolic Analysis

Blood samples for determination of lipolysis, ketogenesis, and other parameters of metabolic adaptation were collected on Day 7 of the experiment. Blood was sampled immediately after morning milking. Vacutainers with clot activator for serum separation (Bexton, Dickinson and Company, Franklin Lakes, NJ, USA) were used for blood collection. After blood clotting, the tubes were placed in a portable refrigerator (at 4 °C) and transported to the laboratory. Blood serum was obtained by centrifugation twice at 3000 rpm for 10 min at room temperature and divided evenly into 3 aliquots. The separated serum was analyzed for biochemical and endocrinological parameters the same day after collection in the Laboratory of Pathophysiology, Department of Veterinary Medicine, University of Novi Sad, Serbia. The following blood biochemical parameters of lipolysis and ketogenesis were determined: non-esterified fatty acids (NEFA) (Cat.No FA115) and beta-hydroxybutyrate (BHB) (Cat.No 21525). Other parameters of the standard metabolic profile in cows were determined: glucose (GLU) (Cat.No 21503), cholesterol (CHOL) (Cat.No 11539), triglycerides (TGC) (Cat.No 11529), total bilirubin (TBIL) (Cat.No 11544), aspartate aminotransferase (AST) (Cat.No 11561), gamma-glutamyl transferase (GGT) (Cat.No 11520), calcium (Ca) (Cat.No 11570), inorganic phosphates (P) (Cat.No 11508), total protein (TPROT) (Cat.No 11553), albumin (ALB) (Cat.No 11573), and urea (Cat.No 11537). Standard kits from Randox (UK) for NEFA and BioSystem (Spain) for other parameters were used on Rayto Chemray 120 spectrophotometer (Rayto Life and Analytical Sciences, China). Endocrinological analyses included determination of insulin (INS) (Cat.No 025260), triiodothyronine T3 (Cat.No 025282), thyroxine T4 (Cat.No 025258), and cortisol (CORT) (Cat.No 025287). An automated immunoassay analyzer TOSOH AIA-360 (Tosoh Bioscience, Tokyo, Japan) was used. The Revised Quantitative Insulin Sensitivity Check Index–β-hydroxybutyrate (RQUICKIBHB) index of insulin resistance was calculated according to the formula [31].

### 2.5. Statistical Analysis

First, the possibility of clustering cows according to LP, EB, MF, and EB×MF when using the values of blood metabolic parameters was examined by principal components analysis (PCA). Unit variance scaling was applied to rows; singular value decomposition with imputation was used to calculate principal components. Prediction ellipses were chosen so that a new observation from the same group was within the ellipse with 95% probability. Classification of cows by MF, LP, EB, and interaction of MF × EB was presented graphically. Online platform for visualizing clustering of multivariate data, CLUSTVIW source: https://biit.cs.ut.ee/clustvis/ (accessed on 22 April 2022), was used.

The influence of MF, LP, EB, and their interaction on selected blood parameters and MY was determined using a general linear model (GLM). Comparison of blood parameter values between groups was made by ANOVA and Tukey’s test. To check assumptions, homogeneity of variance tests, including Bartlett’s test, were employed. Furthermore, Levene´s test for equality of error variances was used. For this purpose, statistical software SPSS, version 22.0 (IBM, Armonk, NY, USA) was utilized.

The correlation between EB, NEFA, and BHB with the studied blood parameters was determined using Pearson’s correlation coefficient and regression analysis, separately for 2X and 3X milked cows that were in PEB and NEB as independent groups. The statistical significance of the connection was determined and the change in the strength of the connection as a function of the group to which the cows belonged was presented graphically. The test used was a z-test for Fisher z-transformed correlation coefficients. The regression line between EB and their blood NEFA and BHB was presented with MF as the covariate. Correlation analysis and regression line figure were carried out with SPSS, version 22.0 (IBM, Armonk, NY, USA). The comparison between correlation coefficients was performed with online calculator http://vassarstats.net/rdiff.html (accessed on 22 April 2022). The strength of the correlation was presented graphically by STHDA http://www.sthda.com/english/rsthda/correlation-matrix.php (accessed on 22 April 2022).

## 3. Results

### 3.1. Clustering of Cows According to Lactation Period, Energy Balance, Milking Frequency, and Their Interactions Based on the Values of Blood Parameters

**Figure 1:**
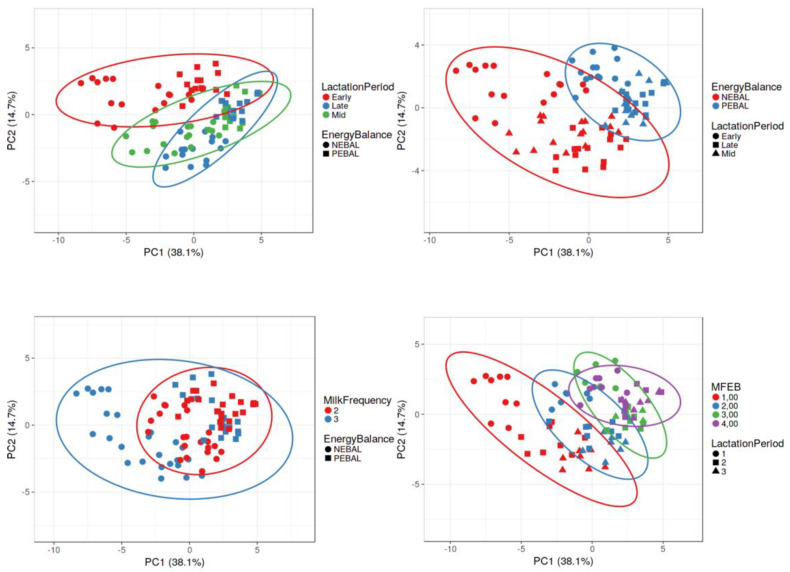
Clustering of cows after the principal component analysis summarizes the information in multiple correlated blood metabolic parameters (MFEB—interaction milk frequency × energy balance; 1-3X milking and NEBAL; 2-2X milking and NEBAL; 3-3X milking and PEBAL; 4-2X milking and PEBAL; NEBAL-negative energy balance; PEBAL-positive energy balance; and PC-principal component 1 and 2 (% of explained total variations)).

Based on the blood biochemical parameters examination, it was possible to distinguish clusters of cows in early lactation in relation to mid and late lactation. Clusters of cows differed partially according to EB, but it was not possible to distinguish cows as a function of MF. However, when cows were classified based on the interaction of EB×MF, the cluster of cows that were milked 3X in NEB stood out the most. It partially overlapped with cows that were milked 2X and were in NEB and did not coincide with clusters of cows that were in PEB. These data indicate that 3X milked cows in NEB exhibit significant metabolic deviations (Figure 1).

### 3.2. Value of Blood Parameters in Function of Lactation Period, Energy Balance, Milking Frequency, and Their Interactions

MF, EB, LP, and MF×EB showed significant effects on metabolic parameters, while the other MF×LP and the 3-way interaction MF×EB×LP did not show significant effects on most blood parameters (Table 3). The values of each blood parameter were studied as a function of EB, MF, LP, and their interactions (Table 4). The LP affected all studied parameters except the values of Ca and P. In early lactation, there are higher values of NEFA, BHB, AST, GGT, TBIL, UREA, and CORT and lower values of GLU, CHOL, TGC, TPROT, ALB, INS, RQUICKIBHB, T3, and T4 compared with other periods. Higher values of NEFA, BHB, AST, GGT, TBIL, and CORT, and lower values of GLU, Ca, INS, and T4 were found in cows milked 3X compared with 2X. The EB showed an impact on all tested parameters except for Ca. Cows in NEB had higher values of NEFA, BHB, AST, GGT, TBIL, UREA, and CORT and lower values of GLU, CHOL, TGC, TPROT, ALB, P, INS, RQUICKI, T3, and T4. The interaction of MF × EB affected all selected parameters except Ca, ALB, UREA, and T4. Cows milked 3X in NEB had higher levels of NEFA, BHB, AST, GGT, TBIL, and CORT and lower values of GLU, CHOL, TGC, TPROT, P, INS, RQUICKIBHB, and T3 compared with cows milked 2X in NEB and cows in PEB. The MF×EB×LP interaction was significant for INS and T3, so that INS and T3 concentrations were lower in 3X milked cows in NEB in early lactation compared with those in PEB, other LPs, and 2X milked cows.

### 3.3. Correlation between Energy Balance, NEFA, BHB, and Other Blood Parameters in Double- and Triple-Milked Cows in Positive and Negative Energy Balance

**Figure 2:**
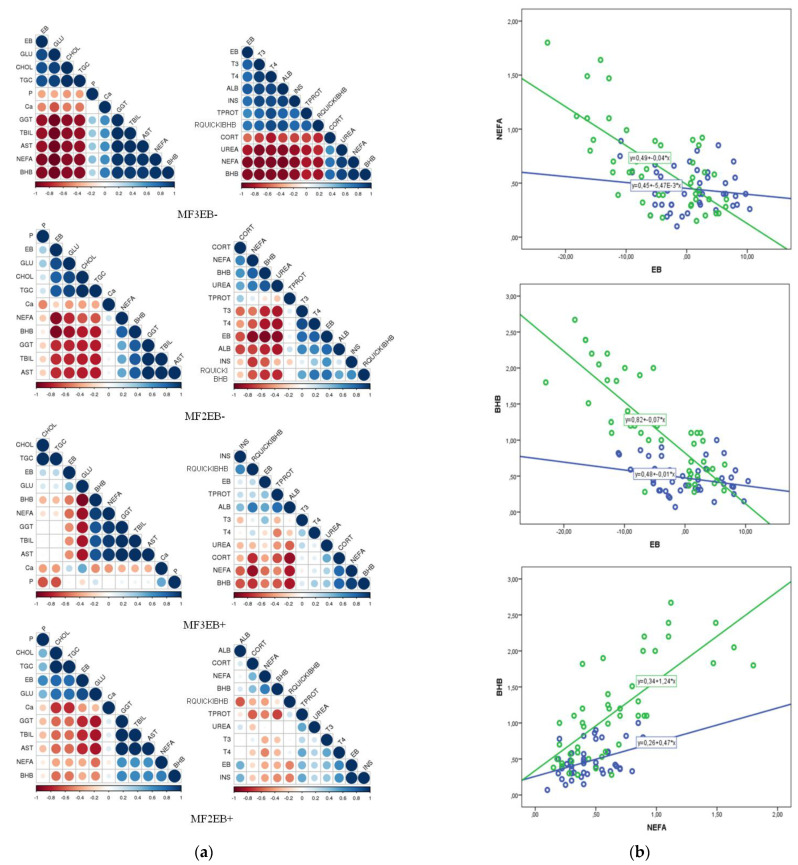
(**a**) Graphical depiction of correlation between energy balance (EB) and blood parameters in four clusters of cows: (MF3EB-)-3X milked in negative EB; (MF2EB-)-2X milked in negative EB; (MF3EB+)-3X milked in positive EB; and (MF2EB+)-2X milked in positive EB; progression from white to dark blue dots—increasing positive correlation; from white to dark red dots-increasing negative correlation; size of dots and intensity of color represents the intensity of correlation; (**b**) Regression line between energy balance (EB, MJ NEL/day), non-esterified fatty acids (NEFA, mmol/L) and beta-hydroxybutyrate (BHB, mmol/L) in 3X (green line) and 2X (blue line) milked cows.

Correlations between EB, NEFA, and BHB and blood parameters were different among cows depending on MF and EB. Very strong correlations were found in cows milked 3X in NEB, and then decreased so that correlations were weaker in cows milked 2X in NEB, even weaker in cows milked 3X in PEB, and many correlations were completely lost in cows milked 2X in PEB (Figure 2a). When we compared the correlation coefficients, we concluded that EB, NEFA, and BHB were more significantly correlated with metabolic parameters in cows milked 3X in NEB (R = 0.78–0.95, *p* < 0.01) compared with the group of cows milked 2X in NEB (R = 0.56−0.79, *p* < 0.01), as well as in relation to the group of cows milked 3X (R = 0.35−0.59, *p* < 0.05) and 2X (R = 0.06−0.31; non-significant) which were in PEB. NEB led to a significantly higher correlation of metabolic parameters with EB, NEFA, and BHB in cows milked 3X compared with the other study groups. Certain parameters, such as Ca and P, showed no relationship with EB, NEFA, and BHB regardless of the group of cows. In cows milked 3X, lipolysis and ketogenesis (higher NEFA and BHB) were much more pronounced with decreasing EB, and ketogenesis (increase in BHB) was positively correlated with lipolysis (increase in NEFA) (Figure 2b).

The relative effect of EB, NEFA, and BHB on the parameters varied and depended on the animal group. In cows in NEB, the value of GLU, CHOL, TGC depended significantly more on the value of EB. In cows milked 3X in NEB, the values of TBIL, AST, and GGT were significantly more dependent on EB compared with other groups of cows. The values of TPROT and ALB were significantly more dependent on EB in cows in PEB, while the value of UREA was more dependent in cows in NEB. INS was significantly more dependent on EB in cows milked 2X in PEB, while the value of T3 was significantly more dependent on EB only in cows milked 3X. RQUICKIBHB positively correlated with EB in cows in NEB. CORT decreased with increasing EB value, which was most pronounced in cows milked 3X in PEB, while there was no effect in cows milked 2X in PEB. The values of GLU, CHOL, TGC, TPROT, ALB, INS, T3, and T4 decreased and the values of AST, GGT, TBIL, UREA, and CORT increased with the increase of NEFA and BHB.

The dependence of TGC, TBIL, AST, GGT, and CORT on NEFA is higher in 3X milked cows regardless of EB, while the dependence of GLU, UREA, INS, and T4 on NEFA was higher in cows in NEB, regardless of MF. RQUICKIBHB was more dependent on NEFA in cows in PEB. The dependence of the selected blood parameters on BHB was consistent, regardless of the EB and the MF, with the dependence decreasing in cows milked 2X in PEB compared with other groups of cows.

## 4. Discussion

### 4.1. Milking Frequency and Energy Balance Adaptation

PCA has shown that blood profiles are more uniform when more factors by which cows were categorized were included. This suggests that we have identified some important features of the data and there may be dependencies, which were determined by the GLM to support this clustering. This preliminary result confirms that it is necessary to include factors such as lactation (LP, MY) and EB in the experimental model to fully understand the metabolic adaptation in cows milked 2X and 3X per day and their productive and metabolic characteristics and differences. In this experiment, cows were assigned to NEB and PEB in double and triple milking groups. MF showed a significant effect on MY, and ECM and no effect on % fat, protein, or lactose. These results agree with the previously reported results that milking 3X leads to an increase in MY with little or no change in milk content [32]. There are several mechanisms that explain the increased MY, such as the increase in the number of mammary epithelial cells, their decreased apoptosis, increased cell activity, and the absence of lactation inhibitors [33]. Decreased feed intake leads to an increase in milk fat [34], and this tendency was also observed in the cows in our experiment.

The value of EB is affected by MF, so that NEB is significantly more pronounced in cows milked 3X than those milked 2X, while in the group of cows in PEB, there is no significant difference between 2X and 3X milked cows. Feed efficiency for milk production (ECM: DMI) was better in 3X milked cows in NEB, compared with 2X milked cows in PEB. This finding agrees with the previously reported results. Namely, ad libitum feeding of 3X milked cows could fail to meet energy requirements [8]. Nutrients have been shown to preferentially flow into milk production regardless of the higher rate of tissue catabolism [3], and increased feed efficiency for milk production occurs only when tissues (liver, muscle, fat, etc.) are ready to provide organic matter to support MY [35]. All this suggests that NEB was the result of increased MY because more dietary energy was directed into the udder to produce milk; therefore, feed efficiency for milk production was higher. MY largely depends on the precursors that reach the mammary gland, and GLU is one of the main precursors [36,37]. In cows selected for high MY, GLU mainly enters the udder, and the body meets its energy needs from its fat reserves [38]. In cows that are milked only once per day, the uptake of GLU and acetate is significantly lower compared with cows that are milked more often [39]. The existence of insulin-independent receptors for GLU in the udder allow this process. In parallel, insulin resistance with reduced INS production and reduced sensitivity of adipose tissue to INS was confirmed in cows during NEB [21]. Deficiency of INS as the main lipogenic hormone and reduced sensitivity of adipose tissue to INS lead to increased lipolysis and ketogenesis and unfavorable metabolic adaptation [40], with an increase in NEFA and BHB, which are the main indicators of NEB and poor metabolic adaptation. From all this, we conclude that the value of EB in 2X and 3X milked cows depends primarily on MY, which activates lipolysis and ketogenesis and further metabolic adaptation.

### 4.2. Milking Frequency, Energy Balance, and Metabolic Adaptation

Metabolic and endocrinological profile results show that NEB, early lactation, and 3X milking affect metabolic parameters, leading to an increase in NEFA, BHB, AST, GGT, TBIL, UREA, and CORT and decreases in GLU, CHOL, TGC, TPROT, ALB, INS, RQUICKIBHB, T3, and T4 compared with PEB, 2X milking, and late lactation, with significant MF×EB interaction. This finding is a direct consequence of energy redistribution and homeorhesis in cows milked 3X in early lactation, in NEB. A similar result was found in experiments with feed restriction in cows [41]. The relationship between the above parameters with EB, NEFA, and BHB shows that the relationships are strongest in 3X milked cows in NEB, and that they are practically absent in 2X milked cows in PEB. Our results are in line with previous results given by Wathes et al. [42] and Djoković et al. [24]. In the study by Wathes et al. [42], correlation was examined as the function of parity and lactation, and correlations were found to depend on these two factors. MY was positively correlated with NEFA and BHB and negatively with INS, and relation strength decreased in later lactation. Djokovic et al. [24] found that correlations between metabolic and endocrinological parameters depended on the value of EB of the cow, since many correlations were lost when the EB value was excluded (when calculating the partial correlation). They also found a constant negative correlation between NEFA and BHB with the values of INS, T3, and T4.

NEFA and BHB are high-value biomarkers of NEB, and an increase in their value increases the risk of developing metabolic diseases [43,44]. NEFA is considered a better predictor of NEB [43]. The relationship between NEFA and BHB shows a weak to moderate positive correlation [45,46,47], with which our results agree. Their growth and correlation depend on MF. Thus, NEB was found to cause more intense lipolysis and more intense ketogenesis in cows milked 3X than in a cow milked 2X. The intensity of ketone formation is thought to depend more on the conversion of fatty acids to ketone bodies and less on the process of β-oxidation [48]. Since NEFA is a predictor of NEB, this could be an explanation for the differences in their correlation and the slope of the curve as a function of MF.

Clustering of cows according to NEFA and BHB significantly affects values of blood metabolic parameters and productivity in dairy cows [49,50,51]. Values of NEFA above 0.5 mmol/L and BHB above 1.2 mmol/L were found in cows with metabolic stress load [50]. These cut-off values are higher in our research, so a specific metabolic adaptation to increased lipolysis and ketogenesis was expected. Reduced DMI and increased GLU utilization for milk production led to lower GLU concentration [52]. Hyperketonemia is negatively correlated with GLU in dairy cows [53], and negative correlation between NEFA and GLU was found in a goat model [47]. Low GLU concentration obstructs INS secretion, lipid mobilization, and ketogenesis and predisposes to insulin resistance [54]. Functional status of hepatocytes significantly depends on degree of lipid mobilization [49]. There is a negative correlation between NEFA and CHOL, and their ratio can be used to estimate hepatic lipidosis [55]. Increased lipid uptake in hepatocytes and limited fat transport from the liver with consequently higher accumulation of triglycerides are the main reasons for reduced concentrations of CHOL and TGC in the blood of cows [56] in the function of lipolysis and ketogenesis. All this leads to the formation of fatty liver within the ketosis–fatty liver syndrome, which leads to hyperbilirubinemia [57]. AST activity was higher in cows with higher ketogenesis, due to hepatocyte load with lipids, catabolism of proteins, and use of their amino acids for gluconeogenesis [58]. Cows with high NEFA and AST are more likely to be culled in herds [59]. Protein metabolism is also affected by EB, lipolysis, and ketogenesis. Urea concentration depends on feed intake, and its determination by NEFA is only at the level of 14% [60]. Reduced concentration of TPROT and ALB is a consequence of decreased biosynthetic function of the liver due to higher ketogenesis in hepatocytes [61]. Xu et al. [62] found reduced protein synthesis in cows with a clinical form of ketosis. Gluconeogenic pathway dominantly uses amino acids when supply of GLU is insufficient [63]; therefore, glucogenic amino acids concentrations increase during hyperketonemia in cows [64]. Decreased concentration of Ca is connected to high concentration of NEFA in cows during early lactation [65,66]. Elevated NEFA with reduced concentration of Ca predisposes cows to diseases in early lactation [67,68]. Decreased concentration of P in blood was found in hyperketonemic cows with low concentration of INS and higher concentration of NEFA [69], and P is related to INS and adipose tissue response during an intravenous glucose tolerance test [70].

Our results, especially in early lactation, are consistent with previous findings. Loiselle et al. [71] found that the increase in NEFA and BHB concentrations were greater in cows milked 2X than in cows milked 1X, serum GLU concentration decreased but remained higher in cows milked 1X, and serum Ca on Day 4 and serum P on Days 4 and 5 were higher in cows milked 1X. Increasing MF is associated with higher MY and metabolic changes during the early postpartum period [72]. Andersen et al. [10] found 19% higher BHB values and 6% lower GLU values in cows milked 3X compared with cows with lower MF. Similarly, plasma NEFA and BHB were lower, and GLU was higher in cows milked 1X [73].

### 4.3. Milking Frequency, Energy Balance, and Endocrine Adaptation

Our results show that cows milked 3X in NEB have higher concentrations of CORT and lower concentrations of INS, T3, and T4 hormones, which is consistent with findings in cattle in the peripartum period. INS concentration decreases, and insulin resistance increases during feed restriction in cows [39] and also during early and pick lactation [74,75]. NEFA is associated with insulin resistance by inducing changes in post-receptor signaling and decreasing the density of GLUT4 [76]. RQUICKI is strongly influenced by NEFA in early lactation [77]. RQUICKIBHB negatively correlates with NEFA and BHB [24]. Weber et al. [78] showed significantly reduced RQUICKI indices in a group of cows with higher triglycerides accumulation in the liver, which occurs during lipolysis, ketogenesis, and NEB. RQUICKI decreases during feed restriction in mid and late lactation or increases in early lactation [38]. In this study, it was found that in cows milked 3X in NEB, RQUICKIBHB decreased, while in cows milked 2X in NEB, it slightly increased, so this index also showed duality in the change of values in our case.

Huszenicza et al. [79] showed a negative correlation between NEFA and thyroid hormones in cows in early lactation. In hyperketonemic cows, blood levels of thyroid hormones T3 and T4 were significantly lower around calving than in healthy cows [80,81]. LP, MY, and nutrient intake significantly affect thyroid hormone concentrations [82]. A negative correlation was found between MY and T4 hormone [83]. All the above results support our finding that there are lower concentrations of thyroid hormones in cows milked 3X in NEB.

Serum CORT concentrations were higher in cows in NEB than in cows in PEB during feed restriction [84], but the response of CORT to ACTH is lower during early lactation and in high yielding cows [85], which may lead to the conclusion that lactation is a type of stress and depletes CORT reserve in the adrenal cortex. One of the most common metabolic diseases that accompanies lactation is ketosis in cows, and a lower concentration of CORT was found in cows with ketosis [86], which confirms that reduced cortisolemia is the result of exhaustion of adaptive abilities of cows. Elevated levels of CORT in 3X milked cows could be related to the increased needs of the organism for gluconeogenesis [87] and the production of higher amounts of GLU, which is necessary for milk production. It is especially important that glucocorticoids enhance the lipolytic effect of glucagon, epinephrine, and growth hormone [88]. Growth hormones are known to be responsible for galactopoiesis and lactation persistence [89], so increased CORT levels in 3X milked cows due to higher MY could be a logical finding. Higher CORT levels in cows may be related to more frequent exposure to the automatic milking system, although results in this area are contradictory [90,91]. All this could be related to higher CORT values and their association with MF and metabolic adaptation to NEB, lipolysis, and ketogenesis. All mentioned physiological and pathophysiological mechanisms support the results obtained in terms of the intensity of metabolic changes in the function of EB, lipolysis, and ketogenesis in double- and triple-milked cows.

### 4.4. Limitation of Study

In our trial, EB was obtained by calculation as an individual EB, based on a detailed analysis of rations, body weight, and MY and predicted DMI on farm. This procedure was weighed as feasible, due to the agreement between calculated and real EB in cows [92,93,94]. EB depends on DMI, and it has been found that there is inter- and intra-individual variability in DMI intake in cows [95]. The main limitation of this study is the calculative determination of DMI based on the formula. On commercial farms, DMI is calculated on the basis of the expected average MY of the group. Cows in our experiment received the same ration in different amounts depending on productivity. If the ration is the same, the feed bunk space/cow is sufficient (exceeds a minimum of 0.8 m) and the DMI of cows in the group is similar. Cows in our study were observed during eating, so it is clear that there will be a discrepancy between the DMI and the actual nutrient requirements because cows did not have the same body weight and MY, and they were not even in the same LP. The obtained values of EB according to the calculated DMI proved to be a good model in this case because such classification led to deviations in the value of metabolic parameters. Therefore, regardless of the limitation regarding the absence of individual measurement of DMI, it can be concluded that the use of the formula for DMI and EB according to NRC was sufficient for this study because the outcome variables from the metabolic profile showed significant deviations as a function of classification according to EB and MF. Reproducibility of this study is possible if the experiment is conducted on the farm where management is changing from 2X to 3X MF and cows are divided in two groups based on MF. It is of great importance to select uniform cows with similar production characteristics, body condition and size, determine a sufficient number of individuals, and to use standards that reduce the risk of imprecision and bias https://www.ncbi.nlm.nih.gov/books/NBK519366/table/cerguideassess.tab3/ (accessed on 22 April 2022). The study can be conducted on different farms, but then it is necessary to include the influence of the farm and ration in the model.

## 5. Conclusions

On the basis of metabolic and endocrine profile parameters, we conclude that cows milked 3X in NEB represent a specific metabolic cluster. Homeorhesis is more pronounced in cows milked 3X, as reflected by increased lipolysis, ketogenesis, decreased glycemia and insulinemia, and increased hepatocyte load. Metabolic and endocrinological parameters are determined mainly by the values of EB, NEFA, and BHB in 3X milked cows in NEB compared with other categories of cows. The change in the value of metabolic parameters per unit EB and NEFA depends on milking frequency and EB, while the change in the value of metabolic parameters as a function of BHB is constant regardless of milking frequency and EB of cows. These results confirm the peculiarity of metabolic adaptation in cows with increased milking frequency, which is characterized not only by differences in the concentration of metabolites but also in their interactions.

## Figures and Tables

**Table 1:** Chemical composition of the total mix ration components (% in dry matter (DM)) according to NRC (2001).

Component *	Dry Matter, %	Crude Protein, %	Crude Fat, %	Raw Ash, %	NEL **, MJ/kg	NDF, %	ADF, %	Lignin,%	Ca, %	P, %	kg DM	CONCENTRATE, %
1	43.6	7	3.3	3.1	6.73	32.2	17.5	1.4	0.28	0.26	8.72	/
2	33	17.3	2.5	10.4	5.43	44.1	33.4	6.6	1.39	0.36	1.65	/
3	84.3	19.6	1.4	11.5	5.8	40.9	29.9	5.5	1.37	0.3	1.94	/
4	26.9	10	7	4	7.34	48	23.1	5	0.91	0.09	1.08	/
5	21.8	28.4	5.2	4.9	7.15	47.1	23.1	4.7	0.35	0.59	0.96	/
6	88.1	9.4	4.3	1.5	8.41	9.5	3.4	0.9	0.04	0.3	5.38	50.33
7	91	12.4	2.2	2.9	7.78	20.8	7.2	1.9	0.06	0.39	0.61	5.52
8	90.3	37.8	5.4	7.4	7.36	29.8	20.5	9.5	0.75	1.1	0.92	8.40
9	89.1	49.9	1.6	6.6	8.91	14.9	10	0.7	0.4	0.71	0.87	8.07
10	92.2	37	1.4	7.7	5.78	40.3	30	9.5	0.48	1	1.44	12.85
11	90.3	32.6	1.7	6.5	6.57	36.1	22.1	8.3	0.4	0.83	0.55	5.02
12	100	0	0	100	0	0	0	0	39.4	0	0.13	1.07
13	100	0	0	100	0	0	0	0	0	0	0.06	0.49
14	100	0	0	100	0	0	0	0	0	0	0.06	0.49
15	98	0	0	100	0	0	0	0	0	0	0.05	0.41
16	99	0	0	100	0	0	0	0	32.8	0	0.18	1.48
17	99	56	0	85.1	0	0	0	0	12	16	0.12	0.99
18	99	283	0	0.03	1.9	0	0	0	6.8	0	0.18	1.48
19	100	0	0	100	0	0	0	0	0	0	0.02	0.16
20	100	0	99	0	32.64	0	0	0	0	0	0.39	3.21
21	52.89	17.01	4.76	6.81	7.24	28.13	16.53	3.1	0.97	0.45	25.3	100.0

* 1-Corn silage, multiple grains, 2-Haylage of alfalfa, average, 3-Hay of alfalfa, average, 4-Silane beet noodles, 5-Beer trope, fresh, 6-Corn, grain, 7-Barley grain, 8-Oil shot rapeseed, 9-Soybean meal 44%, 10-Sunflower meal 33%, 11-Extruded flaxseed, 12-Livestock chalk, 13-Livestock salt, 14-Baking soda, 15-MgO, 16-Premix, 17-Phosphozel, 18-Zenural (urea), 19-Bentonite (Mycotoxin adsorbent), 20-Dairyfat c 16, and 21-Chemical composition. ** Abbreviations: NEL-net energy of lactation, NDF-neutral detergent fiber, and ADF-acid detergent fiber.

**Table 2:** Milk yield, chemical composition of milk, and dry matter intake (DMI) used for classification of selected 3X and 2X per day milked cows in different lactation periods divided into energy balance positive (Pos) and negative (Neg) groups.

Production Parameters	Energy Balance	Cows Milked 3X Daily						Cows Milked 2X Daily					
		Early		Mid		Late		Early		Mid		Late	
		Mean	SD *	Mean	SD	Mean	SD	Mean	SD	Mean	SD	Mean	SD
Milk (kg/day)	Neg	30.7	2.4	43.8	5.03	40.0	5.5	29.7	1.7	37.3	1.7	38.0	3.1
	Pos	25.2	1.83	40.1	1.77	41.3	2.3	25.9	1.6	34.0	2.6	35.1	2.5
Milk Fat (%)	Neg	4.61	0.92	4.46	0.43	4.35	0.44	4.32	0.71	4.48	0.4	4.7	0.51
	Pos	3.57	0.28	3.47	0.26	3.53	0.31	3.70	0.4	3.8	0.2	3.63	0.2
Milk Protein (%)	Neg	3.65	0.35	3.89	0.49	3.95	0.50	3.40	0.1	3.57	0.8	3.96	0.6
	Pos	3.48	0.45	3.95	0.5	2.47	0.65	3.73	0.3	3.57	0.7	3.58	0.2
Lactose (%)	Neg	4.93	0.1	5.03	0.2	4.95	0.25	4.94	0.15	5.06	0.2	4.91	0.15
	Pos	4.82	0.12	4.85	0.11	4.78	0.11	4.81	0.2	4.8	0.2	4.73	0.07
Energy-corrected Milk (kg/day)	Neg	34.3	6.03	48.8	5.4	43.9	5.14	31.3	2.97	40.72	2.07	43.32	3.09
	Pos	23.9	1.76	36.47	1.04	37.6	0.96	25.4	2	33.87	1.92	33.97	2.64
DMI Predicted (kg/day)	Neg	19.8	2.28	26.8	1.94	25.51	1.73	19.6	1.1	24.1	0.8	25.3	1.04
	Pos	17.3	1.02	23	0.35	23.41	0.31	18.04	0.75	21.9	0.77	22.19	0.88
Body Weight (kg)	Neg	582.2	15.5	611.2	19.9	619.7	15.5	592.6	17.5	620.2	10.4	629.5	12.6
	Pos	588.3	22.3	605.6	21.5	625.8	16.8	589.5	18.9	611.4	15.2	631.7	14.3
Energy Balance (MJ NEL/day)	Neg	−13.68	4.95	−11.27	4.09	−5.01	2.37	−5.88	2.37	−4.73	1.84	−4.51	2.55
	Pos	1.44	0.92	0.98	0.86	0.9	0.31	1.97	1.5	1.94	1.5	3.31	2.21
Number of Cows	Neg	9		7		8		7		8		7	
	Pos	6		8		7		8		7		8	

* Abbreviations: SD—standard deviation.

**Table 3:** General linear model—influence of lactation period (LP), milking frequency (MF), energy balance (EB), and their interaction on milk production and blood metabolic and endocrine parameters in cows. Table represents determined *p* values.

	MF	EB	LP	MF×EB	MF×LP	EB×LP	MF×EB×LP
Milk_LperDay	0.000	0.000	0.000	0.131	0.033	0.214	0.334
MilkFat	0.925	0.000	0.984	0.077	0.373	0.724	0.030
MilkProtein	0.593	0.000	0.448	0.244	0.004	0.000	0.066
Lactose	0.118	0.000	0.075	0.153	0.450	0.141	0.438
NEFA	0.000	0.000	0.000	0.000	0.000	0.050	0.144
AST	0.001	0.000	0.000	0.001	0.071	0.041	0.179
GGT	0.001	0.000	0.000	0.001	0.072	0.041	0.179
Ca	0.000	0.188	0.527	0.109	0.928	0.183	0.170
P	0.325	0.009	0.431	0.073	0.075	0.389	0.941
TPROT	0.114	0.012	0.269	0.924	0.972	0.198	0.841
ALB	0.815	0.000	0.008	0.001	0.944	0.540	0.103
UREA	0.048	0.000	0.000	0.164	0.280	0.667	0.052
INS	0.265	0.000	0.431	0.034	0.746	0.869	0.001
RQUICKIBHB	0.108	0.000	0.001	0.397	0.158	0.001	0.984
T3	0.012	0.000	0.000	0.000	0.801	0.299	0.019
T4	0.647	0.002	0.000	0.000	0.079	0.012	0.118
CORT	0.142	0.000	0.002	0.010	0.953	0.150	0.223

Abbreviations: non-esterified fatty acids (NEFA), beta-hydroxybutyrate (BHB), glucose (GLU), cholesterol (CHOL), triglycerides (TGC), total bilirubin (TBIL), aspartate aminotransferase (AST), gamma-glutamyl transferase (GGT), calcium (Ca), inorganic phosphates (P), total protein (TPROT), albumin (ALB), insulin (INS), revised quantitative insulin sensitivity check index–β-hydroxybutyrate (RQUICKIBHB), triiodothyronine (T3), thyroxine (T4), and cortisol (CORT).

**Table 4:** Comparison between groups with post-hoc analysis.

Parameter	Energy Balance	3X Milking						2X Milking					
		Early		Mid		Late		Early		Mid		Late	
		Mean	SD	Mean	SD	Mean	SD	Mean	SD	Mean	SD	Mean	SD
NEFA (mmol/L)	Neg	1.29 ^a^	0.32	0.68 ^b^	0.17	0.43 ^c^	0.20	0.60 ^b^	0.19	0.34 ^c^	0.16	0.38 ^c^	0.19
	Pos	0.74 ^b^	0.17	0.35 ^c^	0.11	0.32 ^c^	0.15	0.59 ^b^	0.21	0.37 ^c^	0.10	0.42 ^c^	0.15
BHB (mmol/L)	Neg	2.15 ^a^	0.29	1.46 ^c^	0.41	1.04 ^e^	0.47	0.67 ^d^	0.21	0.36 ^f^	0.24	0.50 ^d^	0.17
	Pos	0.89 ^b^	0.30	0.64 ^d^	0.16	0.41 ^f^	0.11	0.51 ^d^	0.26	0.30 ^f^	0.15	0.50 ^d^	0.15
GLU (mmol/L)	Neg	1.94 ^a^	0.19	2.20 ^c^	0.21	2.56 ^d^	0.14	2.27 ^c^	0.08	2.73 ^f^	0.29	2.88 ^f^	0.22
	Pos	2.41 ^b^	0.11	2.54 ^d^	0.17	2.78 ^e^	0.11	2.33 ^c^	0.11	2.74 ^f^	0.36	3.00 ^f^	0.29
CHOL (mmol/L)	Neg	2.58 ^a^	0.30	2.74 ^a^	0.53	3.63 ^c^	0.65	3.48 ^c^	0.38	4.08 ^b^	0.58	4.51 ^b^	0.63
	Pos	4.08 ^b^	0.99	4.50 ^b^	0.78	4.49 ^b^	1.17	3.13 ^c^	1.08	4.20 ^b^	1.50	4.49 ^b^	1.07
TGC (mmol/L)	Neg	0.09 ^a^	0.01	0.09 ^a^	0.02	0.12 ^c^	0.02	0.12 ^c^	0.01	0.14 ^d^	0.02	0.15 ^d^	0.02
	Pos	0.16 ^b^	0.04	0.17 ^b^	0.03	0.17 ^b^	0.04	0.12 ^c^	0.04	0.16 ^b^	0.06	0.17 ^b^	0.04
TBIL (μmol/L)	Neg	18.6 ^a^	4.79	11.3 ^c^	5.34	6.73 ^e^	2.52	8.86 ^f^	3.40	8.36 ^f^	5.31	5.05 ^e^	1.94
	Pos	9.52 ^b^	3.59	4.11 ^d^	1.42	4.47 ^d^	1.48	8.08 ^f^	1.94	5.63 ^e^	1.72	4.82 ^d^	1.86
AST (U/L)	Neg	96.8 ^a^	24.9	58.8 ^b^	27.8	35.0 ^d^	13.1	46.1 ^b^	17.7	43.5 ^b^	27.6	26.3 ^e^	10.1
	Pos	46.6 ^b^	17.6	20.1 ^c^	6.94	21.9 ^c^	7.25	39.6 ^b^	9.48	27.6 ^e^	8.42	23.6 ^c^	9.11
GGT (U/L)	Neg	32.3 ^a^	8.30	19.6 ^b^	9.25	11.7 ^d^	4.37	15.6 ^b^	5.90	14.5 ^b^	9.20	8.75 ^c^	3.36
	Pos	15.6 ^b^	5.86	6.71 ^c^	2.31	7.30 ^c^	2.42	13.2 ^b^	3.16	9.19 ^d^	2.81	7.87 ^c^	3.04
Ca (mmol/L)	Neg	2.22 ^a^	0.46	2.00 ^a^	0.40	1.86 ^b^	0.44	2.73 ^d^	0.20	2.53 ^d^	0.22	2.66 ^d^	0.29
	Pos	2.18 ^a^	0.25	2.13 ^a^	0.15	2.40 ^c^	0.23	2.60 ^d^	0.33	2.67 ^d^	0.52	2.59 ^d^	0.20
P (mmol/L)	Neg	1.89 ^a^	0.19	1.95 ^a^	0.22	1.72 ^a^	0.49	1.77 ^a^	0.60	1.93 ^a^	0.22	2.04 ^b^	0.18
	Pos	2.22 ^b^	0.47	2.13 ^c^	0.06	2.21 ^b^	0.18	1.82 ^a^	0.41	1.91 ^a^	0.37	2.21 ^b^	0.42
TPROT (g/L)	Neg	57.4 ^a^	5.78	63.5 ^c^	5.98	63.4 ^c^	6.99	65.4 ^c^	6.25	63.8 ^c^	5.48	69.7 ^c^	7.61
	Pos	75.1 ^b^	10.8	77.6 ^b^	6.32	79.9 ^b^	3.92	68.9 ^c^	7.69	76.9 ^b^	4.49	73.9 ^b^	3.29
ALB (g/L)	Neg	23.1 ^a^	3.31	26.9 ^b^	3.55	30.5 ^b^	3.63	25.4 ^a^	4.13	32.3 ^d^	2.94	32.9 ^d^	3.58
	Pos	26.6 ^b^	6.29	38.4 ^c^	4.28	36.0 ^c^	6.29	32.7 ^d^	6.98	33.9 ^d^	4.30	36.2 ^c^	5.19
UREA (mmol/L)	Neg	7.53 ^a^	1.63	4.99 ^b^	1.31	4.42 ^b^	2.02	6.57 ^a^	1.32	5.33 ^b^	1.29	4.32 ^c^	1.25
	Pos	5.35 ^b^	1.22	4.04 ^b^	1.73	4.99 ^b^	1.25	3.74 ^c^	1.19	4.00 ^c^	1.47	4.38 ^c^	1.22
INS (mU/L)	Neg	2.49 ^a^	0.97	4.45 ^b^	0.90	4.01 ^b^	0.70	3.04 ^c^	0.61	3.38 ^c^	1.31	3.14 ^c^	0.69
	Pos	4.42 ^b^	0.67	4.90 ^b^	1.32	5.11 ^b^	0.58	5.14 ^b^	1.25	6.86 ^d^	2.21	7.68 ^d^	1.70
RQUICKIBHB	Neg	0.40 ^a^	0.14	0.46 ^c^	0.04	0.62 ^b^	0.14	0.58 ^b^	0.09	0.65 ^b^	0.08	0.63 ^b^	0.16
	Pos	0.58 ^b^	0.09	0.83 ^d^	0.13	0.71 ^d^	0.10	0.52 ^b^	0.09	0.62 ^b^	0.16	0.54 ^b^	0.06
T3 (nmol/L)	Neg	0.48 ^a^	0.19	0.98 ^b^	0.22	0.95 ^b^	0.42	0.55 ^a^	0.18	0.83 ^c^	0.28	0.81 ^c^	0.42
	Pos	1.05 ^b^	0.32	1.07 ^b^	0.18	0.99 ^b^	0.30	1.14 ^b^	0.26	1.38 ^b^	0.34	1.37 ^b^	0.38
T4 (nmol/L)	Neg	12.1 ^a^	6.49	28.4 ^c^	10.2	29.7 ^c^	11.9	23.2 ^d^	4.04	29.3 ^c^	7.69	38.3 ^b^	15.7
	Pos	37.1 ^b^	9.10	42.9 ^b^	21.6	27.6 ^c^	10.9	36.5 ^b^	9.04	43.7 ^b^	12.9	56.5 ^d^	18.5
CORT (nmol/L)	Neg	27.2 ^a^	4.88	20.8 ^c^	8.73	18.9 ^c^	4.68	16.9 ^c^	2.06	12.9 ^d^	4.89	15.4 ^b^	2.14
	Pos	15.4 ^b^	3.15	12.0 ^d^	3.40	12.2 ^d^	2.99	11.6 ^d^	3.90	8.90 ^e^	3.90	11.3 ^d^	4.79

Abbreviations: non-esterified fatty acids (NEFA), beta-hydroxybutyrate (BHB), glucose (GLU), cholesterol (CHOL), triglycerides (TGC), total bilirubin (TBIL), aspartate aminotransferase (AST), gamma-glutamyl transferase (GGT), calcium (Ca), inorganic phosphates (P), total protein (TPROT), albumin (ALB), insulin (INS), revised quantitative insulin sensitivity check index–β-hydroxybutyrate (RQUICKIBHB), triiodothyronine (T3), thyroxine (T4), and cortisol (CORT). Different superscript means significant difference between means at minimum *p* < 0.05.

## Data Availability

The data presented in this study are available in the article.
